# Supplementary material for: OpenMM 7: Rapid development of high performance algorithms for molecular dynamics
Source: PLoS Comput Biol. 2017 Jul 26;13(7):e1005659. doi: 10.1371/journal.pcbi.1005659 (PMC5549999; doi:10.1371/journal.pcbi.1005659)
Supplement: S1 Source Code — (ZIP) [file pcbi.1005659.s002.zip › OpenMM-7.0.1-Source/docs/api-python/generated/simtk.openmm.openmm.CustomCompoundBondForce.html]

CustomCompoundBondForce — OpenMM 7.0.1.dev-5e86c4f documentation


# CustomCompoundBondForce¶

*class* `simtk.openmm.openmm.``CustomCompoundBondForce`(*\*args*)¶
:   This class supports a wide variety of bonded interactions. It defines a “bond” as a single energy term that depends on the positions of a fixed set of particles. The number of particles involved in a bond, and how the energy depends on their positions, is configurable. It may depend on the positions of individual particles, the distances between pairs of particles, the angles formed by sets of three particles, and the dihedral angles formed by sets of four particles.

    We refer to the particles in a bond as p1, p2, p3, etc. For each bond, CustomCompoundBondForce evaluates a user supplied algebraic expression to determine the interaction energy. The expression may depend on the following variables and functions:

    > - x1, y1, z1, x2, y2, z2, etc.: The x, y, and z coordinates of the particle positions. For example, x1 is the x coordinate of particle p1, and y3 is the y coordinate of particle p3.
    > - distance(p1, p2): the distance between particles p1 and p2 (where “p1” and “p2” may be replaced by the names of whichever particles you want to calculate the distance between).
    > - angle(p1, p2, p3): the angle formed by the three specified particles.
    > - dihedral(p1, p2, p3, p4): the dihedral angle formed by the four specified particles.

    The expression also may involve tabulated functions, and may depend on arbitrary global and per-bond parameters.

    To use this class, create a CustomCompoundBondForce object, passing an algebraic expression to the constructor that defines the interaction energy of each bond. Then call addPerBondParameter() to define per-bond parameters and addGlobalParameter() to define global parameters. The values of per-bond parameters are specified as part of the system definition, while values of global parameters may be modified during a simulation by calling Context::setParameter().

    Next, call addBond() to define bonds and specify their parameter values. After a bond has been added, you can modify its parameters by calling setBondParameters(). This will have no effect on Contexts that already exist unless you call updateParametersInContext().

    As an example, the following code creates a CustomCompoundBondForce that implements a Urey-Bradley potential. This is an interaction between three particles that depends on the angle formed by p1-p2-p3, and on the distance between p1 and p3.

    ```
    CustomCompoundBondForce* force = new CustomCompoundBondForce(3, "0.5*(kangle*(angle(p1,p2,p3)-theta0)^2+kbond*(distance(p1,p3)-r0)^2)");
    ```

    This force depends on four parameters: kangle, kbond, theta0, and r0. The following code defines these as per-bond parameters:

    ```
    force->addPerBondParameter("kangle");
    force->addPerBondParameter("kbond");
    force->addPerBondParameter("theta0");
    force->addPerBondParameter("r0");
    ```

    Expressions may involve the operators + (add), - (subtract), \* (multiply), / (divide), and ^ (power), and the following functions: sqrt, exp, log, sin, cos, sec, csc, tan, cot, asin, acos, atan, sinh, cosh, tanh, erf, erfc, min, max, abs, floor, ceil, step, delta, select. All trigonometric functions are defined in radians, and log is the natural logarithm. step(x) = 0 if x is less than 0, 1 otherwise. delta(x) = 1 if x is 0, 0 otherwise. select(x,y,z) = z if x = 0, y otherwise.

    In addition, you can call addTabulatedFunction() to define a new function based on tabulated values. You specify the function by creating a TabulatedFunction object. That function can then appear in the expression.

    `__init__`(*self*, *numParticles*, *energy*) → CustomCompoundBondForce¶
    :   \_\_init\_\_(self, other) -> CustomCompoundBondForce

        Create a CustomCompoundBondForce.

        |  |  |
        | --- | --- |
        | Parameters: | - **numParticles** (*int*) – the number of particles used to define each bond - **energy** (*string*) – an algebraic expression giving the interaction energy of each bond as a function of particle positions, inter-particle distances, angles, and dihedrals, and any global and per-bond parameters |

    Methods

    |  |  |
    | --- | --- |
    | `__init__`((self, numParticles, ...) | \_\_init\_\_(self, other) -> CustomCompoundBondForce |
    | `addBond`((self, particles, parameters) -> int) | addBond(self, particles) -> int |
    | `addFunction`((self, name, values, min, ...) | Add a tabulated function that may appear in the energy expression. |
    | `addGlobalParameter`((self, name, ...) | Add a new global parameter that the interaction may depend on. |
    | `addPerBondParameter`((self, name) -> int) | Add a new per-bond parameter that the interaction may depend on. |
    | `addTabulatedFunction`((self, name, ...) | Add a tabulated function that may appear in the energy expression. |
    | `getBondParameters`(self, index) | Get the properties of a bond. |
    | `getEnergyFunction`((self) -> std::string const &) | Get the algebraic expression that gives the interaction energy of each bond |
    | `getForceGroup`((self) -> int) | Get the force group this Force belongs to. |
    | `getFunctionParameters`(self, index) | Get the parameters for a tabulated function that may appear in the energy expression. |
    | `getGlobalParameterDefaultValue`((self, ...) | Get the default value of a global parameter. |
    | `getGlobalParameterName`((self, ...) | Get the name of a global parameter. |
    | `getNumBonds`((self) -> int) | Get the number of bonds for which force field parameters have been defined. |
    | `getNumFunctions`((self) -> int) | Get the number of tabulated functions that have been defined. |
    | `getNumGlobalParameters`((self) -> int) | Get the number of global parameters that the interaction depends on. |
    | `getNumParticlesPerBond`((self) -> int) | Get the number of particles used to define each bond. |
    | `getNumPerBondParameters`((self) -> int) | Get the number of per-bond parameters that the interaction depends on. |
    | `getNumTabulatedFunctions`((self) -> int) | Get the number of tabulated functions that have been defined. |
    | `getPerBondParameterName`((self, ...) | Get the name of a per-bond parameter. |
    | `getTabulatedFunction`((self, ...) | getTabulatedFunction(self, index) -> TabulatedFunction |
    | `getTabulatedFunctionName`((self, ...) | Get the name of a tabulated function that may appear in the energy expression. |
    | `setBondParameters`(self, index, particles, ...) | setBondParameters(self, index, particles) |
    | `setEnergyFunction`(self, energy) | Set the algebraic expression that gives the interaction energy of each bond |
    | `setForceGroup`(self, group) | Set the force group this Force belongs to. |
    | `setFunctionParameters`(self, index, name, ...) | Set the parameters for a tabulated function that may appear in the energy expression. |
    | `setGlobalParameterDefaultValue`(self, index, ...) | Set the default value of a global parameter. |
    | `setGlobalParameterName`(self, index, name) | Set the name of a global parameter. |
    | `setPerBondParameterName`(self, index, name) | Set the name of a per-bond parameter. |
    | `updateParametersInContext`(self, context) | Update the per-bond parameters in a Context to match those stored in this Force object. |
    | `usesPeriodicBoundaryConditions`((self) -> bool) | Returns whether or not this force makes use of periodic boundary conditions. |

    `getNumParticlesPerBond`(*self*) → int¶
    :   Get the number of particles used to define each bond.

    `getNumBonds`(*self*) → int¶
    :   Get the number of bonds for which force field parameters have been defined.

    `getNumPerBondParameters`(*self*) → int¶
    :   Get the number of per-bond parameters that the interaction depends on.

    `getNumGlobalParameters`(*self*) → int¶
    :   Get the number of global parameters that the interaction depends on.

    `getNumTabulatedFunctions`(*self*) → int¶
    :   Get the number of tabulated functions that have been defined.

    `getNumFunctions`(*self*) → int¶
    :   Get the number of tabulated functions that have been defined.

    `getEnergyFunction`(*self*) → std::string const &¶
    :   Get the algebraic expression that gives the interaction energy of each bond

    `setEnergyFunction`(*self*, *energy*)¶
    :   Set the algebraic expression that gives the interaction energy of each bond

    `addPerBondParameter`(*self*, *name*) → int¶
    :   Add a new per-bond parameter that the interaction may depend on.

        |  |  |
        | --- | --- |
        | Parameters: | **name** (*string*) – the name of the parameter |
        | Returns: | the index of the parameter that was added |
        | Return type: | int |

    `getPerBondParameterName`(*self*, *index*) → std::string const &¶
    :   Get the name of a per-bond parameter.

        |  |  |
        | --- | --- |
        | Parameters: | **index** (*int*) – the index of the parameter for which to get the name |
        | Returns: | the parameter name |
        | Return type: | string |

    `setPerBondParameterName`(*self*, *index*, *name*)¶
    :   Set the name of a per-bond parameter.

        |  |  |
        | --- | --- |
        | Parameters: | - **index** (*int*) – the index of the parameter for which to set the name - **name** (*string*) – the name of the parameter |

    `addGlobalParameter`(*self*, *name*, *defaultValue*) → int¶
    :   Add a new global parameter that the interaction may depend on.

        |  |  |
        | --- | --- |
        | Parameters: | - **name** (*string*) – the name of the parameter - **defaultValue** (*double*) – the default value of the parameter |
        | Returns: | the index of the parameter that was added |
        | Return type: | int |

    `getGlobalParameterName`(*self*, *index*) → std::string const &¶
    :   Get the name of a global parameter.

        |  |  |
        | --- | --- |
        | Parameters: | **index** (*int*) – the index of the parameter for which to get the name |
        | Returns: | the parameter name |
        | Return type: | string |

    `setGlobalParameterName`(*self*, *index*, *name*)¶
    :   Set the name of a global parameter.

        |  |  |
        | --- | --- |
        | Parameters: | - **index** (*int*) – the index of the parameter for which to set the name - **name** (*string*) – the name of the parameter |

    `getGlobalParameterDefaultValue`(*self*, *index*) → double¶
    :   Get the default value of a global parameter.

        |  |  |
        | --- | --- |
        | Parameters: | **index** (*int*) – the index of the parameter for which to get the default value |
        | Returns: | the parameter default value |
        | Return type: | double |

    `setGlobalParameterDefaultValue`(*self*, *index*, *defaultValue*)¶
    :   Set the default value of a global parameter.

        |  |  |
        | --- | --- |
        | Parameters: | - **index** (*int*) – the index of the parameter for which to set the default value - **defaultValue** (*double*) – the default value of the parameter |

    `addBond`(*self*, *particles*, *parameters*) → int¶
    :   addBond(self, particles) -> int

        Add a bond to the force

        |  |  |
        | --- | --- |
        | Parameters: | - **particles** (*vector< int >*) – the indices of the particles the bond depends on - **parameters** (*vector< double >*) – the list of per-bond parameter values for the new bond |
        | Returns: | the index of the bond that was added |
        | Return type: | int |

    `getBondParameters`(*self*, *index*)¶
    :   Get the properties of a bond.

        |  |  |
        | --- | --- |
        | Parameters: | **index** (*int*) – the index of the bond to get |
        | Returns: | - **particles** (*vector< int >*) –   the indices of the particles in the bond - **parameters** (*vector< double >*) –   the list of per-bond parameter values for the bond |

    `setBondParameters`(*self*, *index*, *particles*, *parameters*)¶
    :   setBondParameters(self, index, particles)

        Set the properties of a bond.

        |  |  |
        | --- | --- |
        | Parameters: | - **index** (*int*) – the index of the bond to set - **particles** (*vector< int >*) – the indices of the particles in the bond - **parameters** (*vector< double >*) – the list of per-bond parameter values for the bond |

    `addTabulatedFunction`(*self*, *name*, *function*) → int¶
    :   Add a tabulated function that may appear in the energy expression.

        |  |  |
        | --- | --- |
        | Parameters: | - **name** (*string*) – the name of the function as it appears in expressions - **function** (*TabulatedFunction \**) – a TabulatedFunction object defining the function. The TabulatedFunction should have been created on the heap with the “new” operator. The Force takes over ownership of it, and deletes it when the Force itself is deleted. |
        | Returns: | the index of the function that was added |
        | Return type: | int |

    `getTabulatedFunction`(*self*, *index*) → TabulatedFunction¶
    :   getTabulatedFunction(self, index) -> TabulatedFunction

        Get a reference to a tabulated function that may appear in the energy expression.

        |  |  |
        | --- | --- |
        | Parameters: | **index** (*int*) – the index of the function to get |
        | Returns: | the TabulatedFunction object defining the function |
        | Return type: | TabulatedFunction |

    `getTabulatedFunctionName`(*self*, *index*) → std::string const &¶
    :   Get the name of a tabulated function that may appear in the energy expression.

        |  |  |
        | --- | --- |
        | Parameters: | **index** (*int*) – the index of the function to get |
        | Returns: | the name of the function as it appears in expressions |
        | Return type: | string |

    `addFunction`(*self*, *name*, *values*, *min*, *max*) → int¶
    :   Add a tabulated function that may appear in the energy expression.

    `getFunctionParameters`(*self*, *index*)¶
    :   Get the parameters for a tabulated function that may appear in the energy expression.

    `setFunctionParameters`(*self*, *index*, *name*, *values*, *min*, *max*)¶
    :   Set the parameters for a tabulated function that may appear in the energy expression.

    `updateParametersInContext`(*self*, *context*)¶
    :   Update the per-bond parameters in a Context to match those stored in this Force object. This method provides an efficient method to update certain parameters in an existing Context without needing to reinitialize it. Simply call setBondParameters() to modify this object’s parameters, then call updateParametersInContext() to copy them over to the Context.

        This method has several limitations. The only information it updates is the values of per-bond parameters. All other aspects of the Force (such as the energy function) are unaffected and can only be changed by reinitializing the Context. The set of particles involved in a bond cannot be changed, nor can new bonds be added.

    `usesPeriodicBoundaryConditions`(*self*) → bool¶
    :   Returns whether or not this force makes use of periodic boundary conditions.

        |  |  |
        | --- | --- |
        | Returns: | false |
        | Return type: | bool |

    `__delattr__`¶
    :   x.\_\_delattr\_\_(‘name’) <==> del x.name

    `__format__`()¶
    :   default object formatter

    `__getattribute__`¶
    :   x.\_\_getattribute\_\_(‘name’) <==> x.name

    `__hash__`¶

    `__reduce__`()¶
    :   helper for pickle

    `__reduce_ex__`()¶
    :   helper for pickle

    `__sizeof__`() → int¶
    :   size of object in memory, in bytes

    `__str__`¶

    `getForceGroup`(*self*) → int¶
    :   Get the force group this Force belongs to.

    `setForceGroup`(*self*, *group*)¶
    :   Set the force group this Force belongs to.

        |  |  |
        | --- | --- |
        | Parameters: | **group** (*int*) – the group index. Legal values are between 0 and 31 (inclusive). |

High performance molecular simulation on GPUs


### Navigation

- Application Layer
- Library Layer
  - Core Objects
  - Forces
  - Integrators
  - Extras

©2015, Stanford University and the Authors.
|
Powered by Sphinx 1.3.1
& Alabaster 0.7.7
|
Page source
